# Treatment of Young Permanent Avulsed Teeth with Multidisciplinary Approach—A Case Report

**DOI:** 10.3390/dj12120380

**Published:** 2024-11-23

**Authors:** Narda Lorena Rivera-Pimentel, Nadia Phenelope Campos-Lara, Oscar Arturo Benítez-Cárdenas, Verónica Méndez-González, Andreu Comas-García, Marlen Vitales-Noyola, Gabriela Torre-Delgadillo

**Affiliations:** 1 Pediatric Dentistry Postgraduate, Faculty of Dentistry, Autonomous University of San Luis Potosi, San Luis Potosí 78290, Mexico; nlrp.2906@gmail.com (N.L.R.-P.); nadia.campos@uaslp.mx (N.P.C.-L.); 2 Department of Oral and Maxillofacial Surgery, Faculty of Dentistry, Autonomous University of San Luis Potosi, San Luis Potosí 78290, Mexico; oscar.benitez@uaslp.mx; 3 Endodontics Postgraduate Program, Faculty of Dentistry, Autonomous University of San Luis Potosi, San Luis Potosí 78290, Mexico; veronica.mendez@uaslp.mx (V.M.-G.); marlen.vitales@uaslp.mx (M.V.-N.); 4 Department of Microbiology, School of Medicine, Autonomous University of San Luis Potosi, San Luis Potosí 78290, Mexico; andreu.comas@uaslp.mx; 5 School of Medicine, Cuauhtemoc University San Luis Potosí, San Luis Potosí 78290, Mexico

**Keywords:** dental trauma, pediatric patient, dentoalveolar, endodontic, rehabilitation

## Abstract

**Background:** Dental trauma very commonly comprises lesions that affect the teeth and their supporting structures. In pediatric ages, the main reasons for dental trauma are falls and accidents at school and while practicing recreative activities and sports. Fortunately, dental avulsions are not common; however, they are key factors in the loss of teeth if the issue is not adequately treated in a quick way, so is very important for parents, teachers, or any person that is present with a child during the accident to have knowledge about dental trauma, specifically regarding avulsed teeth, since the best treatment is reimplantation. **Methods:** In this case of dentoalveolar trauma, concerning two avulsed teeth, it was very interesting that the tooth that was reimplanted more quickly (40 min) had a worse prognosis than the tooth that was reimplanted 2 h later, since the tooth that was reimplanted more quickly was transported in a personal wallet, which was a highly contaminated area, unlike the tooth that was reimplanted later, which was transported in a solution as an alternative means of transport. **Conclusions:** So, it is very important that this type of trauma is adequately managed in an interdisciplinary way across multiple dentistry specialties.

## 1. Introduction

Traumatic dental injuries affect the teeth and their supporting structures, including the pulp and periodontal tissues. These injuries are the second most common dental condition after caries and are a frequent cause of oral emergencies. They are classified into acute or chronic lesions and are particularly prevalent among school-aged children, and can affect temporary or permanent teeth. The prevalence of traumatic dental injuries in primary teeth is 22.7%, while it decreases to 15.2% after permanent dentition [1]; however, these data is very contradictory, since this depends on several factors, such as analyzed population and risk factors, among others. With this in mind, Prieto-Regueiro, B., et al., in 2021, evaluated the prevalence of traumatic injuries in child populations (aged 3–5 years) and found very low percentages (12%) regarding the most frequent lesion, which was crown discoloration (0.4%), followed by crown fracture (0.1%) and avulsion (0.1%), and the most affected tooth was the deciduous upper central incisor [2].

Children, across all age groups, are especially vulnerable to dental trauma, with a higher risk during their first two years of life. This period is marked by increased mobility as they begin to walk, leading to frequent falls. Epidemiological data indicate that about 30% of children over the age of seven sustain traumatic injuries to their permanent incisors; however, these data depend on the analyzed population [3].

Traumatic dental injuries are a leading cause of dental consultations and are considered emergencies requiring immediate medical attention. Of these injuries, the intrusion and avulsion of primary teeth are particularly concerning as they can significantly impact the development of permanent teeth. Dental trauma in growing children can affect multiple aspects of their lives, including chewing, articulation, and esthetics. It can also have broader implications for their mental health, emotional well-being, and social skills. Therefore, it is crucial for dental professionals to be knowledgeable about the prevention, diagnosis, and treatment of dental trauma. Common causes of dental trauma include unintentional falls, collisions, and accidents during recreational activities. Additional risk factors include sports participation, bicycle accidents, violence, car accidents, orthodontic treatments, and certain systemic conditions such as epilepsy, autism spectrum disorders, and heart disease. These injuries are particularly prevalent among children and young adults [3,4,5,6].

Among the main post-traumatic complications of dental injuries are necrosis, pulp infection, pulp space obliteration, a various type of radicular resorption, ankylosis, and damage to the marginal gingiva and surrounding bone [7,8,9,10,11]. The proper management of these complications is crucial to preventing adverse outcomes. However, research indicates that many dentists have insufficient knowledge regarding the prevention and emergency management of dental trauma. This knowledge gap leads to significant variability in the treatment of traumatic injuries and often results in unfavorable outcomes for patients. To address this issue, it is essential to develop an internal clinical practice guideline based on evidence-based recommendations. Such guidelines would assist healthcare professionals in effectively managing dental trauma, ensuring consistent and high-quality care for patients [12,13,14]. The management of dental trauma varies depending on the specific tooth or teeth affected. Key steps include a thorough clinical examination, radiographic imaging, photographic documentation, the evaluation of pulp status, and sensitivity and vitality tests. Treatment typically involves the fast implantation of avulsed teeth, stabilization with splints, and the administration of analgesics and/or antibiotics; however, treatment is individualized by patient, according to the type of tooth, whether a tooth is temporary or permanent, or if a fracture is visible. In addition, the consumption of analgesics depends on whether patient is in pain, and use of antibiotics is indicated as a prophylaxis treatment. In the case of re-implantation, a technique in which the avulsed tooth is reinserted inside the alveolus, several studies mention that the reimplanted tooth has a better prognosis if it is replaced with the first few minutes, since time spent outside the mouth is an important factor in cell viability [15,16,17]. The International Association of Dental Traumatology (IADT) has established guidelines for managing traumatic dental injuries in both children and adults [12]. The IADT recommends follow-up with clinical and radiographic evaluations at 3, 6, and 12 months, and annually thereafter for at least 5 years [12].

The aim of this study is to examine the interdisciplinary management of a pediatric dental trauma case in young permanent teeth. This case is particularly noteworthy because, contrary to the literature which states that an avulsed tooth left out of the mouth for more than 60 min has a lower likelihood of viable periodontal ligament cells, the tooth reimplanted most quickly in this case had the worst prognosis. This was due to the significant resorption that occurred despite the rapid reimplantation.

## 2. Case Presentation

A male patient aged 11 years and 3 months presented to the Pediatric Stomatology Clinic (Pediatric Dentistry Postgraduate, Faculty of Dentistry, Autonomous University of San Luis Potosi, SLP, Mexico) with a dental emergency due to dentoalveolar trauma. The patient had no relevant medical or family history. Following a clinical examination and history-taking, informed consent was obtained from the parents, who also approved the proposed treatment plan, and verbal assent from the patient was acquired. In addition, this work was submitted to the Committee of Ethics and Research for case presentation for publication and was approved with the code CEIFE-041-024. The patient reported a fall at school during recess time, where he tripped over his own feet and fell to the ground, and the impact of fall was on the face, specifically the mouth. Clinical examination revealed the following dental injuries: avulsion in teeth 11 and 22; extrusive dislocation with of enamel fractures and dentin and pulp exposure in tooth 21; and subluxation in tooth 12. The avulsed teeth (11 and 22) were out of the mouth for approximately 40 min to 2 h, and thus reimplantation was not immediate. Tooth 22 was transported in the mother’s wallet, resulting in a dry period of about 40 min. Tooth 11 was initially lost after the accident, but the father was instructed to find it and place it in a transport solution. The tooth was located at the accident scene and placed in a Gatorade^TM^ (lemon-lime flavor) solution, where it was preserved for about 15 min.

**Figure 1:**
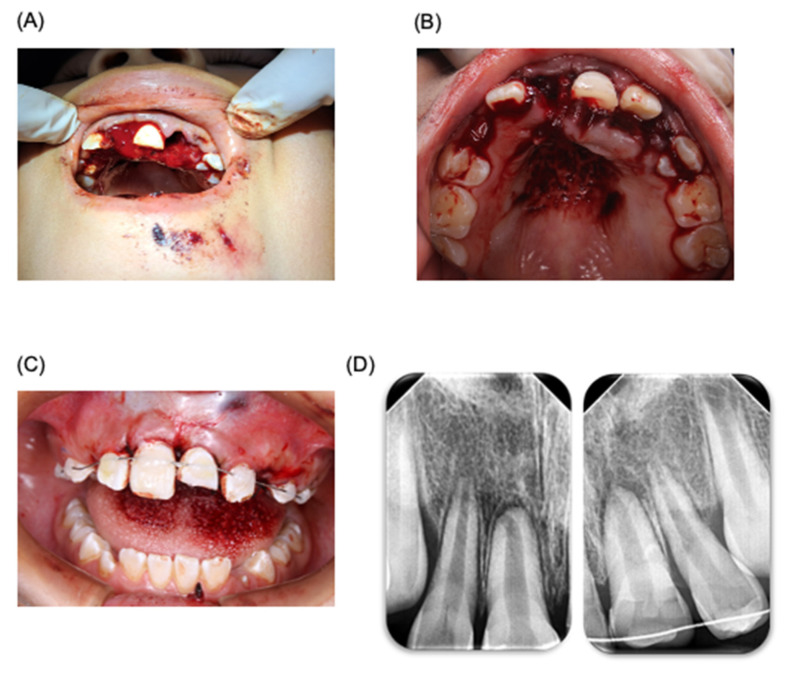
Odontology treatment of patient. Management of dentoalveolar trauma in pediatric patient. (**A**) Photograph of dentoalveolar trauma in pediatric patient. (**B**) Reimplantation of tooth 22. (**C**) Management of trauma using a wire–resin splinting technique. (**D**) Periapical X-ray after splinting treatment.

For the treatment plan, digital periapical radiographs (radiovisograph, Woodpecker Medical Instrument Co., i-sensor H2, Guilin, Guangxi, China) were taken of the trauma area. The area was carefully rinsed with saline solution (PiSA, Guadalajara, Jalisco, Mexico), and anesthesia (articaine 4%, ZEYCO, Zapopan, Jalisco, Mexico) was administered using an infiltrative technique. Curettage of the socket was performed for the reimplantation of tooth 22, applying gentle digital pressure. A digital periapical radiograph was taken to confirm the placement. The same procedure was applied to the other avulsed tooth 11 (Figure 1). It is worth mentioning that the avulsed teeth were rinsed with saline solution before reimplantation. Tooth 21, which had suffered extrusive luxation, was washed with saline solution. A passive splint was made using 0.014 braided wire, extending to teeth 65 and 55 (ten teeth in total, from molars to upper molars). The splinting procedure involved cleaning the area, applying etching acid (3M, St Paul, MN, USA), drying, applying adhesive (3M, MN, USA), and photocuring for 20 s. The splint was placed passively, and fluid composite resin (3M, MN, USA) was used. Periapical radiographs were taken, and the area was sutured with 4-0 silk (ETHICON, Raritan, NJ, USA) using simple stitches (Figure 1). Post-treatment, the patient was prescribed the following medications: amoxicillin/clavulanic acid (400/57 mg/5 mL, every 12 h, for 7 days), acetaminophen (500 mg, every 8 h, for 3 days), ibuprofen (400 mg, every 8 h, for 3 days), and chlorhexidine 0.12% (Lacer, Barcelona, Spain) for 7 days. The patient was told to consume a soft diet for at least 3 weeks. In addition, the case was reviewed by an oral and maxillofacial surgeon regarding the proposed treatment plan (Department of Oral and Maxillofacial Surgery, Faculty of Dentistry, Autonomous University of San Luis Potosi, SLP, Mexico).

**Figure 2:**
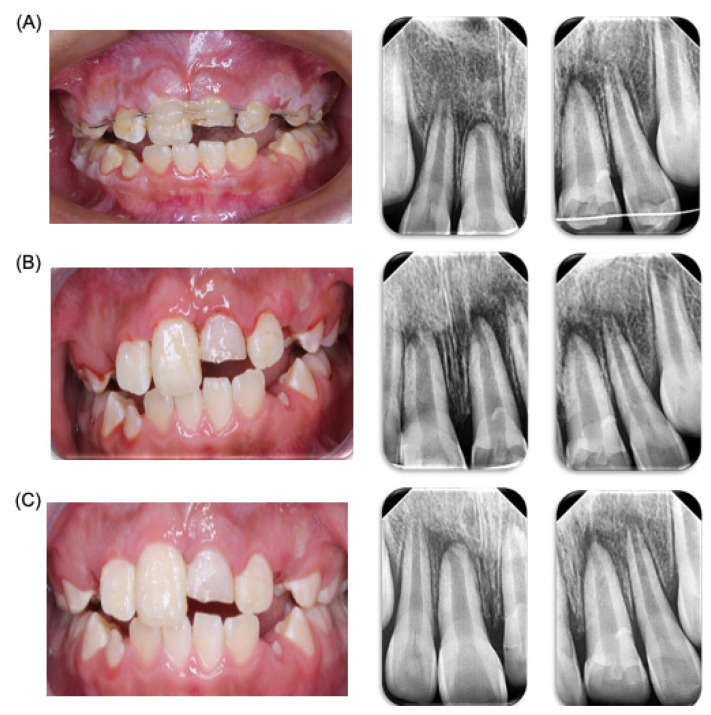
**Dental trauma follow-up.** Control and clinic and radiologic follow-up of patient. (**A**) Clinic and radiological revision one week after accident. (**B**) Clinic and radiological revision two weeks after the accident. (**C**) Clinic and radiological revision due to the new trauma.

A control appointment was scheduled for one week later, with a follow-up for splint removal in two weeks. In the third week, the patient was referred to an endodontist (Clinic of Endodontics, Endodontics Postgraduate Program, Faculty of Dentistry, Autonomous University of San Luis Potosi, SLP, Mexico) for vitality tests. At the follow-up appointment, the patient was asymptomatic but exhibited poor oral hygiene with generalized dental plaque. Instructions were given for the modified Bass brushing technique with a soft-bristled brush. Sutures were removed, periapical radiographs were taken, and the patient was advised to continue with a soft diet. One week later, the patient returned for another control appointment. He was symptom-free and showed improved hygiene. The brushing technique was reinforced, the splint was removed without complications, and additional periapical radiographs were taken. The patient was then referred to the endodontist for further evaluation (Figure 2). Four days later, the patient returned to the pediatric stomatology clinic, reporting additional trauma from a classmate hitting him on the upper anterior teeth. A panoramic X-ray was taken for evaluation. During the physical examination, no new alterations were observed, so continued observation was recommended (Figure 2).

**Figure 3:**
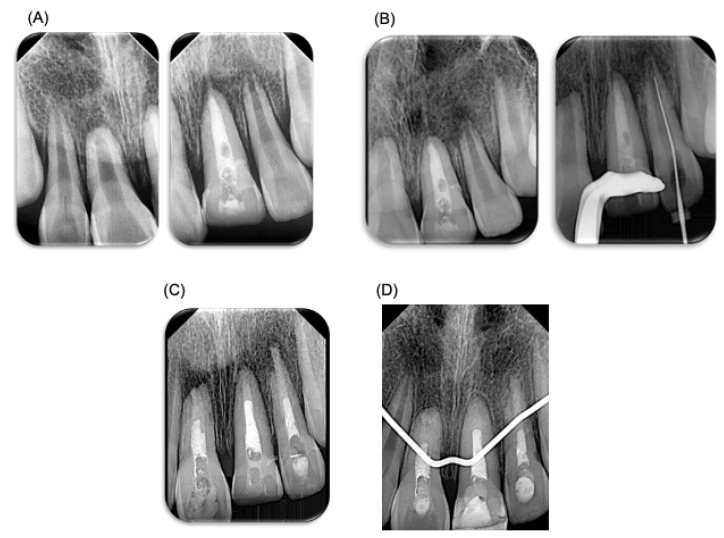
**Endodontic treatment.** Endodontic treatment performed on patient. (**A**) X-ray prior to endodontic treatment performed. (**B**) Control X-ray, 3 weeks after endodontic treatment, where root resorption can be observed. (**C**) X-ray after the performed endodontic treatment. (**D**) X-ray at 3 months, where the apical closure of teeth can be observed (teeth 11 and 21). A Biodentine^TM^ plug was placed. This material has a different radio-opacity than gutta-percha material; therefore, in the X-ray it may look like the endodontic treatment was not performed fully.

A week later, the patient visited the endodontist for evaluation and diagnostic tests, revealing pulp necrosis in tooth 21. Root canal treatment was performed under local anesthesia using mepivacaine 2% with epinephrine 1:100,000 of anterior dental and nasopalatine nerves (Zeyco, Zapopan, Jalisco, Mexico). Absolute isolation was achieved for the access cavity, and odontometry was performed with a K25 file to 19 mm. Protaper Gold rotary systems and K-Files up to a size 90 file were used. The irrigation protocol included 2.25% NaOCl, followed by final irrigation with sonic activation using NaOCl, saline solution, and 17% EDTA (by using three rechanges during 20 s each solution). Root canals were dried with sterile paper points. Intracanal calcium hydroxide medication mixed with water basis was placed, and a temporary restoration with ProRoot (Cavit, 3M ESPE, Seefeld, Germany) was applied (Figure 3). Three weeks after endodontic treatment, tooth 21 was asymptomatic. However, tooth 22 exhibited grade 3 mobility despite being negative on palpation and percussion. In addition, radiographic examination revealed external root resorption in the apical third of tooth 22; therefore, endodontic treatment was performed on tooth 22, carried out following the same specifications. Three weeks later, the temporary filling in tooth 21 was removed (Figure 3). Root canal treatment was also carried out on tooth 11, and endodontic treatment was carried out following the same specifications. Because the teeth had open apices, a biodentine^TM^ (Septodont, Saint-Maur-des-Fossés Cedex, Paris, France) plug was placed for apical sealing.

**Figure 4:**
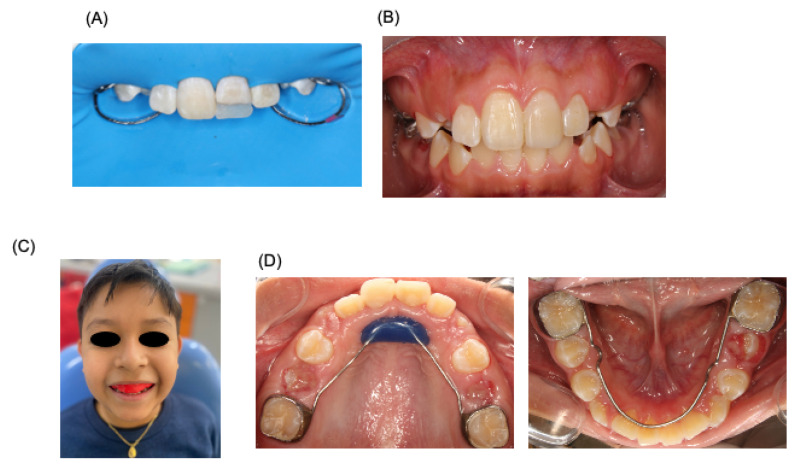
Oral rehabilitation. Treatment of oral rehabilitation of patient. (**A**) Restauration protocol carried out. (**B**) Clinical revision at 7 months after accident. (**C**) Placement of the personalized sports guard. (**D**) Space maintainers placed in the patient’s mouth.

Three months later, the patient was referred to a specialist in esthetics, cosmetics, restorative, and implant dentistry (clinic of prothesis, specializing in esthetics, cosmetics, and restorative and implant dentistry, Faculty of Dentistry, Autonomous University of San Luis Potosi, SLP, Mexico). A direct resin restoration for tooth 21 was performed using a guide and stratification technique with prior waxing (Figure 4). Two weeks later, the patient visited the pediatric dentistry clinic for the placement of a custom sports guard, with follow-up appointments scheduled weekly. A month later, prophylaxis treatment and topical fluoride application were administered. In the subsequent months, a lingual arch and a Nance button were installed to maintain space, with ongoing control appointments. After a month and a half, both the lingual arch and the Nance button were removed (Figure 4), Currently, nine months post accident, the patient continues to be monitored by both pediatric dentists and endodontics, and is currently showing a positive prognosis; however, it will be necessary to evaluate at patient for long time in order be sure of success in treatment.

## 3. Discussion

Tooth avulsion is the complete displacement of a tooth from its socket. While it is a relatively uncommon injury, occurring in 0.5–16% of dental traumas, it most commonly affects the upper central incisors and is more prevalent in male individuals, but this information varies significantly in each analyzed population. The highest prevalence is observed in children aged 7 to 9 years, during the eruption of permanent incisors [18]. At this stage, the roots are not fully formed, the periodontal ligament is laxer, and the surrounding bone is less mineralized, making it less resistant to extrusive forces [18,19]. In our case, the patient was 11 years old, but he did not have an apical closure in the avulsed teeth (teeth 11 and 22) nor the luxated tooth (tooth 21). Tooth avulsion requires immediate medical attention, due to the risk of losing the tooth. When a tooth is forcibly removed, it causes significant damage to the periodontal ligament and cementum, leading to the rupture of the neurovascular bundle and subsequent pulp necrosis. However, teeth with an open apex may still have a chance of revascularization due to their abundant blood supply if reimplanted promptly. In our case, the avulsed and/or luxated teeth did not have an apical closure, and physiological closure was not achieved; therefore, as part of the endodontic treatment, a biodentine^TM^ plug was placed. The success of reimplanting an avulsed tooth depends on several clinical factors, including the time the tooth has been out of the mouth, the condition of the periodontal ligament, the preservation medium used, and the stage of root development. Recommended storage media for avulsed teeth include saliva, Hank’s Balanced Salt Solution (HBSS), coconut water, physiological saline, contact lens solution, milk, isotonic drinks, and green tea, among others [20,21]. Hank’s Balanced Salt Solution (HBSS) is considered the ideal storage medium for avulsed teeth due to its ability to maintain the viability of periodontal ligament cells for extended periods [22,23]. This solution contains essential metabolites and glucose necessary for cell survival, has an optimal pH (6.6 to 7.8) and osmolarity (230–400 mOsmol/kg), and can preserve cells and tissues for up to 24 h without refrigeration. However, its drawbacks include its high cost, limited availability, and short shelf life. In this case, despite a tooth not being placed in a transport medium (tooth 22), the reimplantation was successful. This positive outcome may be attributed to the short time the tooth spent outside the mouth; however, as time passed, this tooth exhibited reabsorption (3 weeks later), which indicates a less positive prognosis and that the placement of a tooth in transport medium is more adequate. For this reason, the endodontist did not initially perform the endodontic treatment but waited for the revascularization process. Since the tooth did not have an apical closure, it was decided that treatment should be postponed until physiological closure, which did not occur, and endodontic treatment had to be performed later, with the placement of a biodentineTM plug. Biodentine^TM^ is a bioceramic cement based on tricalcium silicate, which has good sealing properties and can be used to completely replace dentin, both at the coronal and root levels, as well as for furcation sealing, perforation sealing, retro-filling in apical surgery, apexification, and internal and external resorptions [24,25].

Energy drinks like Gatorade™ have been suggested as alternative storage media for avulsed teeth due to their greater availability and accessibility compared to specialized solutions [22]. Research indicates that energy drinks can preserve periodontal ligament cells better than water, making them a viable option for short-term storage [22]. However, other research suggests that Gatorade™ is not an ideal storage medium. Its low pH can damage cell membranes, impairing cell growth, while its hypertonic osmolarity may cause cells to lose water and die. In this case, one avulsed tooth was stored in Gatorade (tooth 11) and showed a good prognosis, despite being out of mouth longer, in comparison with the other avulsed tooth. Finally, this indicates that while Gatorade™ might offer some benefits for short-term storage, both the duration for which the tooth is out of the mouth and the condition of the storage medium are crucial factors [23]. Further research is needed to clarify these issues.

The immediate reimplantation of the avulsed tooth presents a greater probability of survival of the tooth, and unfortunately, the general public, or even doctors, may lack the necessary skills to perform this procedure. It is of utmost importance to minimize the time of extra-alveolar drying to avoid the death of the periodontal ligament cells that remain on the root surface, since necrosis can occur 30 to 60 min after the avulsion, which directly decreases the chances of success and the good prognosis of dental reimplantation [2]. 

## 4. Conclusions

A thorough patient history, comprehensive clinical examination, and appropriate diagnostic imaging, such as various dental X-rays, are crucial for effectively managing dental trauma. Given the prevalence of dental injuries in children, commonly caused by falls and sports activities, an interdisciplinary approach to healthcare is essential for optimal treatment and patient care. Providing clear information and instructions to patients and their families on how to handle dentoalveolar trauma is vital. Additionally, dentists must stay up to date with best practices and guidelines for managing such injuries to ensure the best outcomes. Given that many traumas that involve the facial region are also treated in hospitals, it is important to note that not only oral health professionals are required for these situations, and medical care must also involve doctors and nurses.

## Data Availability

The original contributions presented in the study are included in the article, further inquiries can be directed to the corresponding author.
